# Patient-reported outcome measures can advance population health, but is access to instruments and use equitable?

**DOI:** 10.3389/fped.2022.892947

**Published:** 2022-10-18

**Authors:** Carolyn F. McCabe, G. Craig Wood, Jennifer Franceschelli-Hosterman, William J. Cochran, Jennifer S. Savage, Lisa Bailey-Davis

**Affiliations:** ^1^ Geisinger Obesity Institute, Danville, PA, United States; ^2^ Population Health Sciences, Geisinger, Danville, PA, United States; ^3^ Nutrition and Weight Management, Geisinger Medical Center, Danville, PA, United States; ^4^ Nutritional Sciences, Center for Childhood Obesity Research, The Pennsylvania State University, University Park, PA, United States

**Keywords:** patient-reported outcome measure (PROM), pediatric care settings, social determinansts of health, healthcare factors, individual-level factors

## Abstract

Patient reported outcome measures (PROM) can engage patients and clinicians to improve health outcomes. Their population health impact may be limited by systematic barriers inhibiting access to completion. In this analysis we evaluated the association between individual parent/child characteristics and clinic factors with parental completion of a locally developed PROM, the Early Healthy Lifestyles (EHL) questionnaire. Participants included parent-child dyads who presented at 14 pediatric clinics for regularly scheduled well-child visits (WCV) prior to age 26 months. EHL items include feeding practices, diet, play time, screen exposure, and sleep. Completion was categorized at patient- (i.e., parent-child dyad) and clinic-levels. Parents completed the 15-item EHL in the patient portal before arrival or in the clinic; ninety-three percent of EHL questionnaires were completed in the clinic vs. 7% in the patient portal. High-completers completed EHL for half of WCVs; low-completers completed at least once; and non-completers never completed. Clinics were classified by EHL adoption level (% high completion): High-adoption: >50%; Moderate-adoption: 10%–50%; and Low-adoption: <10%. Individual-level factors had negligible impact on EHL completion within moderate/low EHL adoption sites; high-adoption sites were used to evaluate infant and maternal factors in association with EHL completion using hierarchical logistic regression. Noncompletion of EHL was significantly associated (*p* < 0.05) with infant use of public insurance (OR = 1.92 [1.42, 2.59]), >1 clinic site for WCV (OR = 1.83 [1.34, 2.50]), non-White birth mother (OR = 1.78 [1.28, 2.47]), and body weight <2,500 grams or gestational age <34 weeks (OR = 1.74 [1.05, 2.90]). The number of WCVs, a proxy for clinic size, was evaluated but was not associated with completion. Findings indicate potential disparities between populations exposed to, completing, and benefitting from these tools.

## Introduction

Patient-reported outcomes (PROs) are any report, direct from the patient without interpretation, of the status of a patient's health condition or health behavior (1). Often, PROs collect self-reported patient data on health-related quality of life; symptoms and symptom burden; and health behaviors (e.g., diet or sleep hygiene) (1). Patient-reported outcome measures (PROM) are the tools by which PROs are measured or collected, and often take the form of a standardize questionnaire delivered to patients in either a condition-specific or generic, routine care setting (2). Although PROM were originally created for use in clinical trials to evaluate treatment efficacy, they are increasingly used to support clinical decision making. PROM encourage the engagement of patients in their healthcare and facilitates patient-provider communication and individualized patient care by healthcare providers (3). PROM have also been successfully implemented in pediatric care settings for disease-specific (4–6) and primary care applications (7). Yet, less is known about the equity of PROM implementation across health systems and patient access to these tools (8). Evidence suggests that barriers to implementation of PROM in clinical settings include technology, stakeholder uncertainty about the, “How” or “Why” PROM would be used, stakeholder concerns about the negative impact of PROM, and competing demands from current clinic workflows (8). However, once PROM are implemented in clinical settings across healthcare systems, it is also unclear whether these barriers to implementation translate to disparities in adoption of PROM and patient completion of the tools.

Social determinants ranging from the structure and systems level (e.g., federal or state regulations), community level (e.g., policies or built environment), institutional level (e.g., schools or healthcare administration), interpersonal level (e.g., support systems), to the individual level (e.g., health behaviors or beliefs) are interconnected and impact the health achievement of individuals (9). Although individual characteristics such as sex, BMI, or age are associated with PROM completion (10, 11), no study has simultaneously evaluated upstream system-level factors and individual-factors associated with PROM completion. In this paper, we performed an observational study with a retrospective chart review to examine individual (parent/child), and clinic factors associated with parental completion of a locally developed PROM, the Early Healthy Lifestyles (EHL) questionnaire, at Geisinger pediatric care clinics between 2016 and 2020. We hypothesize that institutional-level (e.g., clinic size) factors and individual-level factors (e.g., insurance type) will be associated with the completion of PROM.

## Methods

### Early healthy lifestyles (EHL) tool

The EHL PROM is a risk assessment tool comprised of 15-items (e.g., questions) that allow parents or caregivers to self-assess obesogenic behaviors that may increase their child's risk for obesity. The EHL tool was originally created and implemented as part of the WEE Baby Care study—a pragmatic, randomized clinical trial that compared standard pediatric clinical care to a responsive parenting intervention (12). The EHL tool's theory was informed by the Centers for Disease Control and Prevention's Infant Feeding Practices Study II (13, 14), the Intervention Nurses Start Infants Growing on Healthy Trajectories (INSIGHT) trial (15) and Healthy Active Living for Families (HALF) materials (16). The EHL tool assesses the domains of infant feeding and parenting practices: food and beverage intake, sleep hygiene, physical activity, television/media exposure, and soothing practices. As such, the EHL identifies signals across several domains that are aligned with anticipatory guidance to support regulation of eating, sleep, emotion, and activity for healthy development. The EHL is currently available only in English and requires less than five minutes to complete. Standard of care at Geisinger is for the EHL tool to fire two weeks prior to each scheduled well-child visit (WCV) that occurs at a Geisinger clinic for children between birth and 26 months of age. The tool can be completed in the patient portal (MyGeisinger, i.e., MyChart in EPIC® Electronic Health Record) prior to arrival, on a clinic iPad in the waiting room, or in the exam room *via* staff interview. All procedures were approved by the Geisinger Clinic Institution Review Board (IRB).

### Inclusion / exclusion criteria

For this study WCV encounters were limited to those WCVs that occurred at a clinic that was offering the EHL; limited to corresponding time windows of 2016–2020; and the parent or caregiver responded to EHL Q1 with either, “I live with this child and care for him/her regularly” or “I do not live with this child but care for him/her regularly.” Criteria for available pediatric patient data included a baseline measurement whereby the child attended a qualifying WCV at age <3 months / weight and length (or height) were measured; a qualifying WCV while aged 6–15 months with weight and length (or height) measured; and a qualifying WCV while aged 18–26 months with weight and length (or height) measured. All patients (i.e., parent-child dyads) must have had at least 3 WCVs at study clinics.

Completer vs. non-completer definitions were established to ensure equal average dose or opportunity for completion of the EHL tool at qualifying WCVs. An EHL questionnaire was only considered complete if 100% of the questions were answered. Three EHL completion groups were established. A Non-completer completed the EHL zero times at their qualifying WCVs. A Completer was split into low and high categories: Low: completed EHL at 1%–49% of their qualifying WCVs and High: completed EHL at ≥50% of their qualifying WCVs (17). These criteria resulted in the identification of *N* = 4,960 eligible patients. When available, maternal electronic health record (EHR) data was linked with infant EHR using the Births / Deliveries ID's using International Classification of Diseases, 9th Revision (ICD-9-CM) from mother and infant EHR.

Exclusion criteria included: patient visited Geisinger clinics at which EHL was not being administered; attended a WCV outside the time window of 2016–2020; and/or parent/caregiver responded to EHL Q1 with “I do not live with this child regularly and I do not care for him/her regularly.”

### Clinic sites

Clinic sites were conveniently selected for EHL administration, principally because these sites aligned with the WEE Baby Care Study (12) implementation or were identified by clinical leadership as viable implementation sites. All clinic staff are instructed to and evaluated on their performance to close care gaps as a quality initiative. Incomplete PROM are flagged as care gaps to alert check-in staff of the opportunity to improve quality. Thus, if EHL were incomplete when the parent presents at the clinic, then staff were instructed to ask the parent to complete the EHL on an iPad, or if the workflow demands, room the patient and collect the data by interview with staff entry into the EHR. The clinics selected for EHL administration are predominantly clustered in two counties across the Central PA region: Luzerne and Lackawanna Counties and encompass a 40–50-mile radius. For the analysis presented in this paper, which covered the years 2016–2020, *N* = 14 clinics were represented. Some patients (19%) had WCV at >1 clinic location, therefore, a “primary clinic site” was chosen for each patient based on the clinic site at which the most WCVs were completed. The distribution of EHL completion group was reviewed and each primary clinic site was categorized into one of three EHL adoption groups, based on the distribution of EHL completion across all sites: High EHL adoption: sites with >50% high completers; Moderate EHL adoption: sites with 10%–50% high completers; Low EHL adoption: sites with <10% high completers. EHL adoption category was the primary clinic factor under consideration. This clinic factor was used to evaluate which sites had sufficient variability to further explore patient level characteristics.

### Statistical analysis

SAS version 9.4 was used for statistical analysis. To evaluate the association between birth characteristics of the child and maternal characteristics with EHL completion, data from labor and delivery (LD) were used to identify children that had available delivery data and to link the mother to the infant. Of the 4,960 infants described above, there were a total of 2,991 (60%) that were in the LD database including *n* = 1,087 from high adoption sites, *n* = 1,363 from moderate EHL adoption sites, and *n* = 541 from low EHL adoption sites. Chi-square and *t*-tests were performed to compare completer vs. non-completer across demographics and Primary Clinic EHL adoption.

Hierarchical logistic regression (HLR) was used to evaluate the infant and maternal characteristics that were associated with completion of EHL. This model used a random intercept term within PROC GLIMMIX in SAS to account for multilevel correlation introduced by clinic site (18). Individual level factors had negligible impact on EHL completion within moderate/low EHL adoption sites, as such the subset of high-adoption sites were used to evaluate whether infant characteristics, birth characteristics, and maternal characteristics were associated with EHL completion in the HLR.

We first selected items for consideration in the HLR model by using clinic size, infant, birth, and maternal characteristics with *p*-values < 0.20 in unadjusted analysis (19). The items were then re-evaluated with an HLR model that accounted for primary clinic site and items that continued to have *p*-value < 0.20 were retained. Models of infant characteristics were then compared using the cohort of patients that attended high-adoption sites (*n* = 1708) vs. a subset of the cohort that attended high-adoption sites and had complete delivery information (*n* = 1087) in addition to birth characteristics and maternal characteristics. Given that these two models were consistent with one another, we chose to continue with the sub-cohort that had complete delivery data (*n* = 1087) to avoid issue with missing data. Lastly, model building was completed using a forward stepwise approach (after considering both strength of association and scientific plausibility) with the goal of identifying a minimal set of clinical variables that independently predict EHL completion.

## Results

Table 1 presents the distribution of number of completed EHL questionnaires by number of WCVs attended per completer group. The median number of WCV was 7 for non-completers and 8 for both low and high completers. Table 2 describes primary clinic sites by EHL adoption category and the respective proportion of individuals in each completer group. There was an unequal distribution of high completers across primary clinic site, with 5 sites categorized as high EHL adoption (comprised of >50% high completers), 2 sites categorized as moderate EHL adoption (10%–49% high completers), and 9 sites categorized as low EHL adoption (<10% high completers). The number of WCVs over a multi-year period varied across the three categories of adoption although high EHL adopters had the smallest variance (*n* = 241) compared to moderate (*n* = 531) and low (*n* = 827) adopters. The ratio of male to female infants was nearly equal (50.7% male overall and not associated with EHL adoption level, *p* = 0.271) (Table 3). Race/ethnicity was different based on EHL adoption level (*p* < 0.0001). Most participants identified as non-Hispanic/Latino White (73% in High adoption sites, 86% in Moderate adoption sites, 74% in low adoption sites), followed by Hispanic/Latino (15%, 7.5%, 18.3%) and African American (10%, 4.4%, 6.5%). The number of WCV attended also differed by clinic adoption level, whereby nine or more WCV were completed by 27.9% of participants in High adoption sites and 19.5% and 25.9% in Moderate and Low adoption sites respectively. Lastly, use of public insurance also differed by clinic adoption level with the greatest percentage of participants (33.9%) at High adoption sites (Table 3).

**Table 1:** The distribution of number of completed EHL questionnaires by number of WCVs attended per completer group.

		**Number of completed EHL**
		0	1	2	3	4	5	6	7	8	9	10	11	Total
**Number of WCV**	3	6	2	4	1	-	-	-	-	-	-	-	-	13
	4	36	21	18	10	4	-	-	-	-	-	-	-	89
	5	88	69	41	31	31	14	-	-	-	-	-	-	274
	6	149	79	79	101	70	44	19	-	-	-	-	-	541
	7	234	166	177	180	136	124	80	52	-	-	-	-	1,149
	8	281	231	244	230	205	162	125	114	96	-	-	-	1,688
	9	147	116	124	115	100	89	79	64	81	61	-	-	976
	10	41	20	15	18	11	9	15	20	13	12	20	-	194
	11	9	2	6	3	1	2	0	1	2	0	5	2	33
	12	0	0	0	0	1	2	0	0	0	0	0	0	3
	Total	991	706	708	689	559	446	318	251	192	73	25	2	4960

Legend: non-completers (*n* = 991), low completers (*n* = 2,055), and high completers (*n *= 1,914).

**Table 2:** Primary clinic sites by EHL adoption category with the proportion of individuals in each completer group.

Primary Clinic Site	*N*	% Non-completers	% Low completers	% High completers
High EHL adoption				
Clinic A	219	0.5%	5.5%	94.1%
Clinic B	436	0.2%	11.7%	88.1%
Clinic C	294	1.0%	18.7%	80.3%
Clinic D	299	1.7%	39.5%	58.9%
Clinic E	460	4.1%	37.0%	58.9%
Moderate EHL adoption				
Clinic F	1,141	7.5%	60.6%	32.0%
Clinic G	610	24.6%	45.1%	30.3%
Low EHL adoption				
Clinic H	169	36.7%	53.9%	9.5%
Clinic I	949	33.0%	59.1%	7.9%
Clinic J	261	91.6%	8.4%	0.0%
Other smaller clinics	122	92.6%	7.4%	0.0%

**Table 3:** Comparison of selected characteristics of patients by level of EHL adoption at their primary clinic site.

Characteristic	Primary Clinic EHL adoption			*p*-value
		High *N* = 1708	Moderate *N* = 1751		Low *N* = 1501
Sex	Male	51.6%	49.1%	51.4%	0.271
	Female	48.4%	50.9%	48.6%	
Race/ethnicity^a^	White	73.4%	86.4%	74.0%	<0.0001
	Black	10.1%	4.5%	6.5%	
	Hispanic/Latino	15.1%	7.5%	18.3%	
	Other	1.5%	1.6%	1.2%	
# of WCV	3–5	6.9%	4.9%	11.5%	<0.0001
	6–8	65.3%	75.6%	62.6%	
	9+	27.9%	19.5%	25.9%	
Public insurance	Yes	33.9%	24.3%	22.5%	<0.0001
	No	66.1%	75.7%	77.6%	

^a^ Race/ethnicity categories are mutually exclusive (e.g., Latino of any race). All other race categories are non-Hispanic/Latino.

Providing that a patient's opportunity for EHL exposure and completion depended upon clinic adoption level, Low EHL Adoption sites were removed from further analysis. Choosing to only evaluate High and Moderate adoption sites, where patient opportunity to complete the EHL was more consistent, allowed for evaluation of our primary interest in individual characteristics associated with EHL completion. Clinic size was not associated with high completion (*p* = 0.795) when evaluating all sites (regardless of adoption level). Furthermore, when limited to high adoption sites, clinic size remained not associated with high completion (*p* = 0.407). Individual characteristics significantly associated (*p* < 0.0001) with High Completion of EHL at High Adoption sites included infant race/ethnicity, utilizing one primary WCV clinic, and use of private insurance (Table 4). Infant birth weight (*p* = 0.015), maternal parity (*p* = 0.03), maternal age (*p* = 0.0049), maternal race/ethnicity (*p* < 0.0001), and maternal use of private insurance (*p* = 0.0003) were also associated with High Completion of EHL (Table 4).

**Table 4:** Comparison of selected patient characteristics by completion group.

Characteristic		High EHL adoption sites			Moderate EHL adoption sites		
	*N*	% high EHL completion	*p*-value	*N*	% high EHL completion	*p*-value
Infant Sex	Male	881	73.80%	0.462	860	31.20%	0.826
	Female	827	75.30%		891	31.70%	
Infant Race/ethnicity	White	1253	78.70%	<0.0001	1513	32.10%	0.157
	Black	173	63.00%		78	23.10%	
	Hispanic/Latino	257	62.30%		132	31.80%	
	Other	25	72.00%		28	17.90%	
Number of WCV clinics	1	1032	79.30%	<0.0001	1596	31.10%	0.625
	2	596	68.30%		143	35.00%	
	3+	80	60.00%		12	33.30%	
Infant Public insurance	Yes	579	63.60%	<0.0001	425	27.10%	0.026
	No	1129	80.20%		1326	32.80%	
Infant Birth Characteristic		High EHL adoption sites			Moderate EHL adoption sites		
		*N*	% high EHL completion	*p*-value	*N*	% high EHL completion	*p*-value
Gestational age	24–33 weeks	24	54.20%	0.085	35	17.10%	0.218
	34–36 weeks	107	70.10%		104	30.80%	
	37–40 weeks	802	74.70%		1069	32.50%	
	41 + weeks	143	69.90%		150	35.30%	
	unknown	11	-		5	-	
Birth weight	<1,500 g	5	40.00%	0.015	10	20.00%	0.353
	1,500–2,499 g	78	62.80%		73	28.80%	
	2,500–2,999 g	194	77.80%		203	27.10%	
	3,000–3,499 g	410	69.30%		463	31.80%	
	3,500–3,999 g	289	76.50%		382	35.30%	
	4,000 g+	79	76.00%		116	34.50%	
	unknown	32	-		116	-	
Maternal Characteristic		High EHL adoption sites			Moderate EHL adoption sites		
		*N*	% high EHL completion	*p*-value	*N*	% high EHL completion	*p*-value
Parity	≤1	449	73.50%	0.0301	581	38.00%	0.0081
	2	341	73.30%		474	28.10%	
	3	170	75.30%		191	28.90%	
	4	84	65.50%		73	26.00%	
	5	25	60.00%		28	28.60%	
	6+	18	83.30%		16	25.00%	
Maternal age	<20	61	60.70%	0.0049	64	23.40%	0.066
	20–24	279	66.70%		250	26.40%	
	25–29	322	75.80%		443	34.80%	
	30–34	286	75.90%		413	34.90%	
	35+	139	78.40%		61	31.60%	
Maternal race/ethnicity	White	795	78.40%	<0.0001	1208	33.20%	0.116
	Black	106	61.30%		44	22.70%	
	Hispanic	164	53.10%		81	29.60%	
	Other/unknown	22	81.80%		30	16.70%	
Maternal public insurance	Yes	283	64.70%	0.0003	585	29.60%	0.064
	No	804	75.90%		778	34.30%	

^a^ Race/ethnicity categories are mutually exclusive (e.g., Latino of any race). All other race categories are non-Hispanic/Latino.

Table 5 presents the final model results of the hierarchical logistic regression and identifies items significantly associated with the odds of noncompletion of the EHL. These items included infant use of public insurance (OR = 1.92 [1.42, 2.59]), greater than one clinic site used for WCV (OR = 1.83 [1.34,2.50]), non-White birth mother (OR = 1.78 [1.28,2.47]), and birthweight <2,500 grams or gestational age <34weeks (OR = 1.74 [1.05, 2.90]).

**Table 5:** Principal associations of variables that independently predict EHL completion.

Effect	Estimate	SE	OR^a^	95% CI	*p*-value
Intercept	−2.095	0.449	-	-	-
Infant public insurance					
Yes	0.651	0.154	1.92	[1.42, 2.59]	<0.0001
No	Reference				
# of clinic sites used for WCV					
1	Reference				
2+	0.605	0.159	1.83	[1.34, 2.50]	0.0002
Birth mother race/ethnicity^b^					
White	Reference				
Non-white	0.574	0.168	1.78	[1.28, 2.47]	0.0007
Birth weight and gestational age					
BW ≥ 2,500 grams and GA 34–40 weeks	Reference				
BW < 2,500 grams or GA < 34 weeks	0.556	0.259	1.74	[1.05, 2.90]	0.032
GA 41 + weeks	0.374	0.220	1.45	[0.94, 2.24]	0.089

^a^ OR is odds ratio for NOT being a high EHL completer.

^b^ Race/ethnicity categories are mutually exclusive (e.g., Latino of any race). All other race categories are non-Hispanic/Latino.

## Discussion

The goal of this study was to investigate the factors associated with the completion or non-completion of the Early Healthy Lifestyles (EHL) PROM. The integration of the EHL into standard pediatric care and the electronic health record (EHR) across several clinical sites in the Geisinger network allowed for both the compilation of a large cohort of individuals that had the opportunity to complete the EHL and for the linkage of health data for infants and mothers.

Our evaluation of the factors associated with completion and non-completion of the EHL tool was important from an implementation science perspective and from a patient-centered healthcare perspective*.* PROM are a standardized way to collect information directly from patients on their experience, perception, or beliefs relative to a disease or health status (20). With the advancement of health information technology (HIT), PROM can be implemented in easy-to-use formats for patients, often *via* an online patient portal or on a tablet in the waiting room of the clinic. Responses can be integrated into the EHR, allowing for quick scoring and review by providers and implementation into patient-centered care (21).

PROM like the EHL are beneficial to population health when exposure to and utilization of the tools is high. Hence, a main aim of this study was to examine the implementation patterns of the EHL across the clinics at which it was active in the system; benefit from patient and provider use of PROM is highly dependent upon exposure or adoption. Initial analyses revealed that clinic site was significantly correlated with completion of the EHL, whereby clinic “adoption” of the tool varied highly across clinics with specific sites more likely to have non-completers over high completers. We also found that primary utilization of more than one clinic site for WVC was associated with EHL noncompletion. Given the spatial context of clinics in this study, it is possible that dyads might attend more than one clinic for convenience; whether some are closer to home while others are closer to work or childcare (22).

Increasing evidence suggests that healthcare system-level factors including characteristics of providers and clinic staff can influence the implementation of PROM (23). Additional factors impacting adoption of collecting of PRO or barriers to PROM are large clinic size [high burden of implementation with the number of patients seen] (24); clinic leadership biases and belief in value of PROM tool (24); and language barriers—most PROM are written in English and this creates a significant barrier for non-English speakers (3, 25). The two clinics with the highest volume of WCVs, a proxy for clinic size, were in the moderate and low EHL adoption categories, whereas the clinics with high adoption had the smallest volume and variance in WCVs, potentially supporting the premise that large clinic size is a barrier. However, total visits, including a distribution of acute and preventive care visits, the number of staff and type, and operating hours would provide a more comprehensive assessment of whether clinic size affects completion. Prior research has identified associations between staff and clinic factors and the receipt of preventive care (26). Future directions include evaluating the features of high, medium, and low adoption sites that might be contributing to completion. It is important to note, however, that we found no association between clinic size and completion that could have been limited to how we defined clinic size as the number of WCVs before age 2 years.

Alternatively, our data reveal that 6.6% of EHL were completed *via* the MyGeisinger patient portal. Increasingly, studies show that electronic patient portals are underutilized, and this is especially true among low-income and minoritized populations (27). Given that patient portals can be a main or important implementation method for PROM, it is relevant to consider how low rates of registration and broader system-level barriers may incite disparities in PROM completion among these communities. Other factors affecting patient portal use and the low utilization rate include lack of patient education about the portal and inconsistent provider facilitation of portal use (27).

In addition to evaluating system-level factors, as encompassed by EHL adoption level, that were associated with the completion of EHL, we sought to elucidate the individual-level factors that might influence completion. We found that the odds of an individual being a non-completer were highest for: infant utilization of public insurance [a surrogate for low-income households (28)], utilizing more than one clinic site for WCV, non-White birthmothers, and infant birthweight of <2,500 grams or gestational age <34 weeks. Some items simultaneously align highly with social determinants of health factors related to preventive care utilization, continuity of care, and attendance of well-child visits. Hardy et al., demonstrated that in rural communities, preventive care receipt is lower, due in part to provider type, poverty, unemployment, and education levels (29). Wolf et al., also detected that poor attendance at preventive care was associated with mothers and children who were publicly insured and children whose mothers had younger age, greater number of pregnancies and transportation (30). Alternatively, it has also been observed that premature and low-birth weight infants display varied health care use patterns. One study revealed that of infants born at ≤35 weeks gestation, 43% completed the full course of preventive care visits in the first 18 months (31). Furthermore, varied patterns in attendance or completion of health supervision visits among premature (32) and low birth weight (33, 34) infants is associated with under vaccination. Lastly, evidence also suggests that continuity of care (e.g., utilizing the same clinic or providers for care and minimal interruptions in the schedule of care) is linked with higher well-visit attendance and improved receipt of preventive screening (35, 36).

Given that the EHL is administered at WCV, and this population is a rural population, it is relevant to consider how these factors may interact. Previous studies conducted in this region and population have detected high utilization rates of public insurance—in 2018, among WCVs across 55 departments at Geisinger, 33.3% of the children with a mean age of 3.3 years received Medical Assistance. The population included in this study displayed a similar rate of public insurance, of around 34%, across a wider window of time. Ultimately, these findings and concurrent parallels with factors associated with receiving care, suggest a larger issue of inequitable access to PROM. Since individual level factors had negligible impact on EHL completion within moderate/low EHL adoption sites, the subset of high-adoption sites was used in the hierarchical logistic regression to evaluate whether infant and maternal factors were associated with EHL completion. In other words, despite attending WCV at High Adoption sites, individual-level factors had a significant effect on determining completion. Additional research is needed to elucidate the context around individuals making it to care but not completing EHL or other PROM either because it is not offered to them specifically or because individuals chose not to complete the tool for personal, health and/or technology literacy, or other reasons.

In this study we were limited by the completeness of infant and maternal EHR for our investigation of individual-level characteristics. We were unable to evaluate food insecurity because of the high rate of missingness on this pre-visit screener. Furthermore, because not all individuals who receive care at Geisinger have delivered at Geisinger, we were unable to acquire Labor / Delivery information on each mother-infant dyad. Despite these limitations, we were able to compile a sizable cohort of *n* = 1,087 mother-infant dyads. It is also relevant to consider the possible impact that the written language of the EHL had on completion. Some of the clinics we evaluated receive a higher percentage of patients for whom English is a second or non-preferred language—we were unable to determine the extent to which this impacted completion. We plan to elucidate language barriers to PROM completion in future investigations.

By using hierarchical logistic regression to model the association between individual-level factors and EHL completion while controlling for clinic location, this study was able to provide valuable insights into the possible reasons for variable completion of PROM in patient care. These results underscore both individual- and system-level factors that influence completion of PROM and illuminate potential disparities between populations being exposed to, completing, and benefitting from these tools.

## Data Availability

The raw data supporting the conclusions of this article will be made available by the authors, without undue reservation.
